# Exploring Sex-Related Differences in Microglia May Be a Game-Changer in Precision Medicine

**DOI:** 10.3389/fnagi.2022.868448

**Published:** 2022-03-31

**Authors:** Marina A. Lynch

**Affiliations:** Trinity College Institute of Neuroscience, Trinity College, Dublin, Ireland

**Keywords:** sex-related differences, age, Alzheimer’s disease, microglia, inflammation

## Abstract

One area of microglial biology that has been relatively neglected until recently is sex differences and this is in spite of the fact that sex is a risk factor in several diseases that are characterized by neuroinflammation and, by extension, microglial activation. Why these sex differences exist is not known but the panoply of differences extend to microglial number, genotype and phenotype. Significantly, several of these sex-related differences are also evident in health and change during life emphasizing the dynamic and plastic nature of microglia. This review will consider how age impacts on sex-related differences in microglia and ask whether the advancement of personalized medicine demands that a greater focus is placed on studying sex-related differences in microglia in Alzheimer’s disease, Parkinson’s disease and models of inflammatory stress and trauma in order to make true progress in dealing with these conditions.

## Introduction

Several diseases are marked by differences in incidence, symptoms and progression between males and females and sex is an acknowledged risk factor in diseases that are characterized by neuroinflammation, including autism spectrum disorders (ASD) Alzheimer’s disease (AD), Parkinson’s disease (PD), multiple sclerosis and migraine (Morgan et al., 2010; Hanamsagar and Bilbo, 2016; Airas et al., 2018; Lecours et al., 2018; Navarro et al., 2018). This sex-related difference is unexplained at this point although studies have provided detailed descriptions of differences in microglial number and morphology, microglial gene signature, microglial phenotype including electrophysiological properties, and microglial function including phagocytosis and antigen presentation which shift during early life, with age and in different pathologies (Guneykaya et al., 2018; Crespo-Castrillo and Arevalo, 2020).

In the past decade or so, particularly with the development of newer techniques, it has become clear that the simplistic view of microglia being “activated” or not, does not reflect the multiple states that the cells can adopt. Instead, attention must be paid to the stimulus that triggers microglial change and a more precise description of the change that includes not only traditional markers of activation but also cell function. While there is a growing appreciation of the need to address these complexities in current and future studies, a great deal of the literature to this point, including that cited in this review, describes microglia as being activated when they adopt a different morphology, and/or express markers of activation, and/or demonstrate a change in function. Therefore the term “microglial activation” is used here but, as far as possible, qualified with a description of the change described by authors.

## Sex Differences in Microglia Throughout Life

### Embryonic and Early Postnatal Life

A number of groups have highlighted sex-related differences in microglial numbers in the early postnatal period which are greater in several areas of the male brain including cortex and hippocampus at P4 (Schwarz et al., 2012). At P20 no sex-related difference in Iba1^+^ cell numbers were identified (Thion et al., 2018) while by P30, microglial numbers were increased in the brain of female rats (Schwarz et al., 2012). Analysis of markers of microglial activation suggested that female rats generally exhibited a more activated phenotype at P0 and a less activated phenotype at P4 than males. By P30 and P60, microglia had developed processes but a sex-related difference in morphology persisted whereby cells from females had thicker longer processes (Schwarz et al., 2012). It has been proposed that the testosterone surge, which occurs in the early post-natal days, is responsible for the differences in number and activation state of microglia in males and estradiol eliminated this sex-related difference, at least in the pre-optic area in P2 male rats (Lenz et al., 2013).

The evidence also indicates time-related and sex-specific changes in phagocytic function. The number of phagocytic cells is greater in P2 (Nelson et al., 2017) and P8 (Weinhard et al., 2018) females, compared with males, and this was eliminated by treatment with estradiol (Nelson et al., 2017), but the evidence suggests that, by p28, phagocytic function is greater in microglia from males (Weinhard et al., 2018). In primary neonatal microglia, however, basal phagocytic activity was greater in females compared with males (Yanguas-Casas et al., 2018). The sex-related difference in phagocytosis was accompanied by increased expression of phagocytic genes notably *Cd68* and Triggering Receptor Expressed on Myeloid cells (*Trem)2*. The microglia specifically phagocytosed neural progenitor cells and it was proposed that, ultimately, this may mean reduced cell proliferation in females.

Recent studies using RNAseq to describe microglia in embryonic development and early life have reported conflicting findings. On the one hand, bulk RNAseq analysis identified a low number of differentially expressed genes in male and female E18.5 mice but showed that expression of genes associated with apoptosis and the inflammatory response, particularly interferon-stimulated genes, was higher in microglia from E18.5 female mice compared with males (Thion et al., 2018). At a slightly earlier age, E14.5, single cell RNAseq revealed no changes in the microglial transcriptome between male and female mice and no differences were found also at P4/5 (Hammond et al., 2019). Gene Ontology (GO) analysis at P20 revealed that genes described by terms such as “inflammatory response,” “immune response,” “immune system processes,” and “response to lipopolysaccharide (LPS)” were upregulated in microglia from females compared with males and this was interpreted as indicating that microglia are in a more primed state in females at this age (Thion et al., 2018); this is perhaps also reflected in the morphological changes that have been described at this age (Schwarz et al., 2012).

While the sex-related differences and very plastic nature of microglia in embryonic and early life are profound, a full understanding of the impact of these changes in infancy, adulthood and beyond remains a challenge. This is complicated by the knowledge that microglia in different brain areas have different signatures (Bordt et al., 2020), highlighting the need for caution in interpretation of findings derived from bulk analysis of microglia. However there is a good deal of evidence indicating that disturbances in microglial dynamics, arising from stressors including infection, during this period correlates with sex-related differences in disorders like ASD and schizophrenia; several excellent reviews have considered this (Ellman and Susser, 2009; Mccarthy and Wright, 2017; Bilbo et al., 2018; Ardalan et al., 2019; Bergdolt and Dunaevsky, 2019). However microglial activation persists into young adulthood (Suzuki et al., 2013) and inflammatory changes have been confirmed in the postmortem brain of individuals with ASD (Li et al., 2009; Tsilioni et al., 2019) suggesting that these ongoing changes may also contribute to ASD, as do environmental factors.

Autism spectrum disorders is heritable and genetic analysis has identified disruption in genes that control protein synthesis (De La Torre-Ubieta et al., 2016). A recent study reported that increasing protein synthesis, by overexpressing the translation initiation factor eIF4E in microglia, resulted in ASD-like behaviors in male mice but not females (Xu et al., 2020). The increase in proteins synthesis triggered a shift from the homeostatic state, altered microglial morphology and reduced motility which, the authors suggested was responsible for the higher spine density, reduced synapse size and altered synaptic function. The important role of microglia in sculpting the central nervous system (CNS) and neurons in particular suggest that alterations in their function such as these may significantly contribute to the neuroanatomical differences that have been described in ASD. Identifying the causes of ASD is not simple but pursuing a greater understanding of sex-related differences in microglial dynamics, in the hope that strategies for reducing the impact of the disorder might be identified, should be a focus.

### Sex Related Differences in Microglia Persist in Adult Animals

**FIGURE 1:**
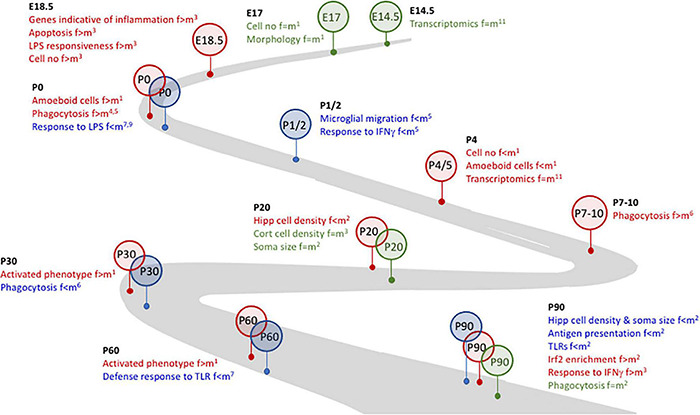
Microglial plasticity is characterized by dynamic changes in early life. Several changes in microglia during development and early life are sex-related. For example. Whereas no marked differences (identified in green) between females and males were identified at E14.5 and E17, at E18.4 changes that were indicative of inflammatory microglia were evident in female, but not male, mice and cells from female mice were more responsive to LPS. By P0, microglia from female mice were more phagocytic and amoeboid (identified in red) but were less responsive to LPS than male mice, suggesting that microglia from female mice may be desensitized to at least some inflammatory stimuli. At P1/2, microglia from male mice (identified in blue) were more motile and more responsive to IFNγ. The timeline to P90, as indicated, is marked with sex-related differences in microglia number and/or function and/or inflammation. References: ^1^Schwarz et al., 2012; ^2^Guneykaya et al., 2018; ^3^Thion et al., 2018; ^4^Nelson et al., 2017; ^5^Yanguas-Casas et al., 2018; ^6^Weinhard et al., 2018; ^7^Hanamsagar et al., 2017; ^8^Villa et al., 2018; ^9^Loram et al., 2012; ^10^Gal-Oz et al., 2019; ^11^Hammond et al., 2019.

Sex-related differences in microglial phenotype endure into adulthood. Guneykaya et al. (2018) reported that microglial density, and cell body volume, were increased in hippocampus of 13-week-old male mice compared with females, while the opposite is the case in 3-week-old mice. This group also reported that microglia from male mice had higher antigen-presenting properties, as indicated by increased expression of MHCII, whereas phagocytic activity of microglia was similar in males and females. Marked sex-related differences in gene expression were identified in microglia prepared from hippocampus and cortex and GO analysis showed that, in hippocampus, overexpression of genes in males was linked with the terms “defense response to bacteria,” “insulin receptor pathway,” and “glia cell differentiation,” whereas genes related to the terms “GABA and glutamate receptor activity,” “ubiquitin protein expression,” and “magnesium iron transport” were overexpressed in females (Guneykaya et al., 2018). In contrast, proteomic analysis determined that interferon regulatory factor (Irf)3 was enriched in microglia from female mice (Guneykaya et al., 2018) consistent with the finding that these cells are more responsive to interferon (IFN) activation (Thion et al., 2018), and this was also suggested by RNAseq analysis in isolated microglia (Gal-Oz et al., 2019). One interpretation of this is that the differential alertness of the female immune system renders it less vulnerable to pathogen-associated molecular pattern (PAMPs) but more reactive to other stimuli that could induce an excessive inflammatory response, an argument that has been used to explain the neuroinflammation that seems to be at the heart of the pathogenesis of some neurodegenerative diseases. In short, responsiveness of microglia to stimuli appears to be sex-dependent in adulthood as it is in earlier life, and the evidence indicates that the plasticity and dynamic nature of these cells persists with time. Some of these sex-related changes are illustrated in Figure 1.

RNAseq, carried out on microglia from pooled brain samples of slightly older, 3 month-old mice, revealed that genes expressed in brains from male mice reflected inflammatory processes (Villa et al., 2018) broadly agreeing with the findings summarized above. In this study, genes from female brains were more associated with morphogenesis, development and cytoskeletal organization suggesting that tissue repair is a particular function of microglia from females. Interestingly, the reparative phenotype that typified microglia from female mice persisted when transplanted into the brain of male mice following ischemic injury (Villa et al., 2018).

### Evidence of Sex-Related Differences in Microglia With Age

While there is a wealth of information relating to age-related changes in microglial activation, phenotype and function and associated increases in inflammatory mediators, to date there has been limited focus on evaluating sex-related differences that occur with age.

Analysis of changes in microglial numbers with age fails to provide a consensus perhaps because of the likelihood of brain area-specific changes, even in subfields of particular structures. For instance, no age-related change was observed in the hippocampus (Vanguilder et al., 2011) but, when area CA1 was assessed, an age-related decrease in cell was observed (Hayakawa et al., 2007). Sex is also a variable since the numbers of microglia in CA1 and dentate gyrus of aged females mice was greater than young female mice and aged male mice (Mouton et al., 2002).

**FIGURE 2:**
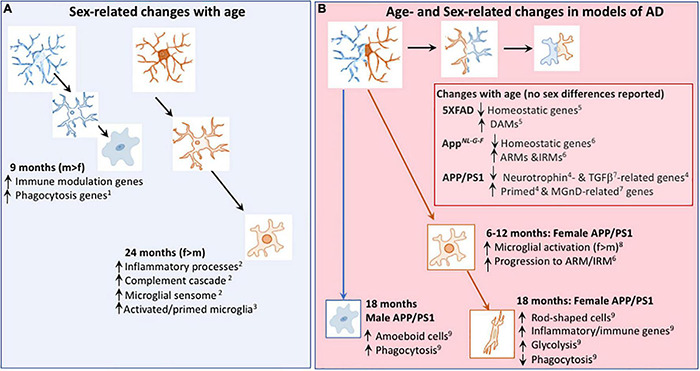
Age- and Sex-related changes in microglia in age and models of AD. **(A)** Microglial phenotype changes with age in a sex-dependent manner. Transcriptomic analysis revealed that genes indicative of immune modulation and phagocytic function are upregulated in 9-month-old male, compared with female, mice^1^. At 24 months of age, transcriptomic analysis indicates that microglia from female mice exhibit greater upregulation of genes including those indicative of inflammatory processes and primed microglia than microglia from male mice^1,3^. **(B)** Changes in the microglial transcriptome that occur with age in models of AD include downregulation of genes that are described as homeostatic^5,6^ and genes that modulate neurotrophins^4^ and TGFβ^7^, together with upregulation of genes that are described as DAMs^5^, ARMs^6^, IRMs^6^, primed^4^ and MGnD-related^7^; sex-related changes were not reported in these studies. From 6 months of age, there was evidence of a change in expression of microglial markers^8^ and a faster transition toward the microglial genotype described as ARMs/IRMs in female APP/PS1 mice compared with males^6^. Microglia from 18 month-old female APP/PS1 mice were glycolytic and exhibited upregulation of genes that are indicative of inflammatory/immune processes^9^ whereas amoeboid and phagocytic microglia were increased in 18 month-old male APP/PS1 mice compared with females^9^. DAM, disease-associated microglia; ARM, Activated response microglia, IRM, interferon response microglia; MGnD, microglial neurodegenerative phenotype. References: ^1^Kodama et al., 2020; ^2^Mangold et al., 2017; ^3^Kang et al., 2018; ^4^Holtman et al., 2015; ^5^Keren-Shaul et al., 2017; ^6^Sala Frigerio et al., 2019; ^7^Krasemann et al., 2017; ^8^Gallagher et al., 2013; ^9^Guillot-Sestier et al., 2021.

Sex-related differences in gene expression in microglia have also been identified. Mangold et al. (2017) reported that age-related changes occur earlier, and are more profound, in hippocampus prepared from female mice compared with males. Transcriptomic analysis in hippocampal tissue from 3, 12 and 24 month-old mice, interrogated by Ingenuity Pathway Analysis (IPA), identified marked upregulation in microglia-specific genes that are indicative of inflammatory processes, components of the complement cascade and the microglial sensome, in tissue from aged female mice (Mangold et al., 2017; Figure 2A). Analysis of changes in microglia isolated from 24-month-old animals revealed that there was a significantly higher expression of *Spp1*, *Gpnmb*, *Lgals3*, *Apoe*, *Ccl3*, *Clec7a*, and *Ccl4* in cells from females compared with males (Kang et al., 2018), genes that identify activated/primed microglia (Holtman et al., 2015).

In contrast, single cell RNAseq in younger, 9-month-old, mice identified marked enrichment of some of these genes and others involved in immune modulation and phagocytosis (e.g., *Lpl, Cst7, Spp1, Tyrobp, Cd9*, and *Cd63*) in males compared with females (Kodama et al., 2020). Two clusters of genes were increased in cells from females; one of these was enriched in homeostatic genes like *P2ry12, Tmem119, Hexb*, and *Sall1* but the second was enriched in genes that have been reported in microglia from models of AD (Keren-Shaul et al., 2017; Sala Frigerio et al., 2019) and multiple sclerosis (Jordao et al., 2019) and these included *Apoe, Ms4a7, Klra2, Clec12a*, and *Mrc1.* Thus there are apparent contradictions in the literature describing age-related microglia but, as in the case of cell numbers, it is likely that sex and brain area significantly contribute to the differences, as well as methodological factors like tissue preparation and analysis.

In humans, aging is marked by changes in gene expression profiles that are brain area-specific with perhaps the most pronounced changes identified in the hippocampus and entorhinal cortex (Berchtold et al., 2008). These authors reported sex-related differences in gene expression in frozen unfixed tissue obtained from individuals aged 20–99 years. In males, the greatest changes occurred in the 6th and 7th decade of life while the most significant changes observed in females were after the age of 80. Genes related to inflammation and immune function were upregulated in tissue from both males and females but the upregulation was markedly greater in females mimicking the sex differences in inflammation in mice (Mangold et al., 2017) and the previous observation that upregulated genes in the aged brain were mostly of glial origin (Erraji-Benchekroun et al., 2005).

It is interesting that, although mRNA expression of estrogen receptor (ER)α and ERβ was unchanged with age in hippocampus of male and female mice, ER transcriptional activity, using the estrogen response element (ERE)-Luc reporter mouse, indicated age-related decreases in several brain areas including hippocampus that were exacerbated by ovariectomy in female mice (Benedusi et al., 2012). The authors described increases in expression of inflammatory markers that accompanied these changes and suggested a causal link between the age-related decrease in ER activity and neuroinflammation. Ovariectomy also increased the expression of several markers of microglial activation in rats (Sarvari et al., 2012) some of which were also increased in post-menopausal women.

## Sex Differences and Microglia: Neurodegenerative Disease

### Sex Differences in Alzheimer’s Disease

Sex differences is a feature of several diseases, including neurological diseases. In the case of AD, the prevalence of the disease in individuals over 65 years is 2–3 times greater in women than men and sex-related differences in pathology and behavior have been reported. For instance, hippocampal atrophy is worse and progresses more rapidly in women than men (Sinforiani et al., 2010; Ardekani et al., 2016), while semantic memory, specifically naming items, is also reported to be more profoundly affected in female AD patients compared with males (Laws et al., 2016). These authors also reported a similar sex-related difference in four other cognitive domains, non-semantic memory, episodic memory, verbal and visuospatial memory. In contrast to AD, a recent metanalysis revealed that there was no significant difference in incidence or prevalence of mild cognitive impairment (MCI) in females compared with males (Au et al., 2017) although a significant increase in prevalence of non-amnestic MCI in females was identified.

The reasons for sex-related bias in AD remain to be identified but sex chromosomes are clearly a factor and it has been reported that X chromosome loss in female (Yurov et al., 2014) and Y chromosome loss in males (Dumanski et al., 2016) were both associated with increased risk of AD. Other contributory factors are sex hormones and genotype (Guo et al., 2021), and there is little doubt that the influence of hormones on AD risk is significant given the well-rehearsed neuroprotective effects of estrogens in aging and injury which have been comprehensively reviewed (Zarate et al., 2017; Duncan, 2020; Kovesdi et al., 2020).

In the context of genes, it is known that the ϵ4 allele of the apolipoprotein E4 gene (*APOE* ϵ4) increases the risk of developing AD to a greater extent in women than men (Altmann et al., 2014) but *ACE* (Crawford et al., 2000) and *BDNF* (Li et al., 2017) have been reported to be female-specific risk genes for AD, while variants in *LINC00290, MPO, NGFR, SERPINB1, TFAM, and ZBTB7C* also confer increased risk of disease in females (Gamache et al., 2020; Prokopenko et al., 2020). Variants in *RELN* confers greater risks in males (Feher et al., 2015), while a sex-specific epistasis between variants of *WWC1* and *TLN2* was observed in male AD patients and this translated into identification of a novel protein-protein interaction that may reflect destabilization of actin filaments (Gusareva et al., 2018). The list of sex x genotype interactions is growing as family based designs coupled with whole genome-sequencing becomes the norm and the emerging evidence emphasizes the importance of studying sex-specific differences in disease causation with the prospect of ultimately identifying sex-specific therapies, but perhaps also considering genotype. In this regard, it is interesting that intranasal administration of insulin to MCI and AD patients improved cognition, particularly *APOE* e4-negative males, but not exert any effect in females (Claxton et al., 2013). The importance of the age x sex x *APOE* has been emphasized in a recent study; age exerted a particularly striking effect on the *Trem2*/*Tyrobp* gene network impacting on microglial phenotype/function while *APOE* markedly *Serpina3n* gene expression that ultimately exerts control over inflammatory and complement cascades (Zhao et al., 2020).

Although there is no effective treatment for AD or for MCI, animal studies continue to offer potential therapeutic avenues including insulin (Mao et al., 2016) and donepezil (Buccafusco et al., 2003). As recently reviewed (Honarpisheh and Mccullough, 2019) and, although not entirely consistent, there is some evidence that women AD patients may be more sensitive to both donepezil and rivastigmine than men, and that there may be sex-related differences in response to memantine and statins. As pointed out in the review, there is a paucity of data addressing sex-related analysis of drug efficacy in AD.

### Microglia and Sex Differences in Alzheimer’s Disease

#### Sex-Related Differences in Differentially Expressed Genes in Microglia: Evidence From Human Studies

Recent studies have attempted to identify a microglial gene signature in healthy aging as well as in disease using the established microglial marker, TMEM119 and a cell-mapping tool, *CellMapper*. Thirty microglia-specific genes not previously identified were found to be enriched in several areas of the healthy human brain, including in areas that are susceptible to AD; these included *TREM2*, *TLR7*, *CSF2RAC3*, and *C1QB* (Bonham et al., 2019). These authors determined that there was marked dysregulation of microglial genes in human tissue from AD patients, particularly in temporal cortex, parahippocampal gyrus and inferior frontal cortex. The changes were more profound in females compared with males in some (e.g., temporal cortex and cerebellum), but not all (e.g., in parahippocampal gyrus, inferior frontal gyrus), brain areas.

Using single nucleus RNA sequencing, isolated nuclei from prefrontal cortex of individuals with amyloid pathology, generated > 80,000 droplet-based single nucleus RNA-seq profiles in identified neurons and glia. Whereas the majority of differentially-expressed genes (DEGs) were downregulated in neurons, they were generally upregulated in glia, including microglia; many were specific to AD and not identified in microglia prepared from samples of aged control individuals (Mathys et al., 2019). Perhaps predictably, upregulation of genes transcripts involved in inflammatory processes and immune activation was identified in AD samples and included genes linked with higher risk of developing AD including *TREM2* and *PICALM*. Other microglial genes that were upregulated overlapped with those identified in mouse models of AD (see below) including *CD74*, *APOE*, *LPL*, and complement and TLR family members, though species-specific differences were identified. These authors assessed sex-related differences, which revealed that AD-associated cell subpopulations were enriched in samples from females and this included one category of microglia. Sex-related differences in other cell types were also found; for example, significant transcriptional activation in oligodendrocytes correlated with pathology in males while pathology in females was associated with a downregulation of gene activity in neurons (Mathys et al., 2019).

#### Sex-Related Differences in Differentially Expressed Genes in Microglia: Evidence From the Study of Animal Models

Differentially-expressed genes have also been identified in mouse models of AD and, using single cell RNAseq, microglia with specific disease-associated signatures have been identified in 5XFAD mice. These so-called disease-associated microglia (DAM) are typified by a downregulation of genes indicative of microglial homeostasis for example *Tmem119*, *P2ry12*, and *Cx3cr1*, and an upregulation of genes recognized as risk factors in AD like *Apoe*, *Tyrobp, Trem2, Ctsd*, and *lpl* (Keren-Shaul et al., 2017).

Analysis of microglia prepared from WT and App*^NL–G–F^* mice of different ages identified clusters of cells expressing different genes depending on age and genotype. A key finding was that the population of activated response microglia (ARMs) were markedly increased in aged App*^NL–G–F^* mice compared with young mice. Furthermore the switch from a homeostatic signature toward the signature identified in ARMs occurred more rapidly in female App*^NL–G–F^* mice compared with males (Sala Frigerio et al., 2019). This earlier sex-related change is consistent with previous findings suggesting that markers of microglial activation, amyloid deposition and behavioral changes all occur earlier in female, compared with male, mice (Gallagher et al., 2013).

A somewhat similar switch from a homeostatic to an activated microglial profile, described as microglial neurodegenerative phenotype (Krasemann et al., 2017), has been identified in other animal models of AD including APP/PS1 mice (Holtman et al., 2015; Krasemann et al., 2017). Induction of these cells was triggered by engulfment of apoptotic neurons resulting in upregulation of several genes indicative of activation including *Apoe*; this upregulation was suppressed in APP-PS1:Trem2^–/–^ mice highlighting the importance of the TREM2-ApoE pathway in the switch from homeostasis (Krasemann et al., 2017). In a separate study, a particularly striking change in an ApoE-driven network converging on CCL3 and CCL4 was observed in aged mice, APP/PS1 mice and AAV-Tau^*P*201*L*^ mice and transcriptomic analysis revealed upregulation of *Cst7*, *Itgax*, *Gpnmb*, *Clec7a*, *Lpl*, *Lgals3*, *Apoe*, and *Spp1*, as well as *Ccl3* and *Ccl4* in these 3 cohorts (Kang et al., 2018). While these studies did not assess sex-related differences, a recent study from this lab demonstrated increased expression in genes described as ARMs/DAMs in microglia from female APP/PS1 mice compared with male APP/PS1 mice (Guillot-Sestier et al., 2021; Figure 2B).

While TREM2 plays an important role in controlling *Apoe* expression in microglia, multiple other roles have been ascribed to it. It controls glial survival and death, regulates chemotaxis and, in models of AD, phagocytic function and cell responses to inflammatory stimuli, and it also controls accumulation of microglia around plaques (Jay et al., 2017). With respect to the latter, it has been proposed that microglial-plaque association may be protective because forming such a barrier may reduce extension of Aβ fibrils from the plaque and thereby limit or prevent neuronal damage (Zhao et al., 2017). The TREM2-dependent interaction between plaques and microglia is affected by sex, at least in 5xFAD mice with *APOE3* knock-in. In these mice, Iba1 plaque coverage and plaque size was markedly reduced in female mice but there was no sex-related difference with *APOE4* knock-in (Stephen et al., 2019). Interestingly TREM2 expression in plaque-associated microglia was about three times greater in male 5xFAD mice with *APOE3* knock-in, compared with females and, in these mice, amyloid pathology was reduced. Consistently, TREM2ko in APP/PS1 mice increased plaque load in 6–7-month-old female mice but no changes were observed in male mice (Meilandt et al., 2020).

In assessing the roles that microglia play in AD models, the focus has been largely on models of amyloidosis. However transcriptomic analysis has identified similar changes in microglia in APP/PS1 mice and in a tauopathy model, AAV-Tau^*P*301*L*^ mice (Kang et al., 2018). While tau pathology was similar in 9-month-old males and females in this model, sex-related differences in miRNA-seq revealed changes accompanying tau pathology that were significantly greater in males compared with females (Kodama et al., 2020). The impact of miRNA on the microglial transcriptome was substantial since knockout of Dicer increased the numbers of amoeboid microglia and increased enrichment of genes involved in inflammation and phagocytosis, including *Spp1, Ccl6, Lpl, Il1b*, and *Cst7* and these changes were much more profound in microglia from males compared with females. The data point to a significant role for miRNAs in modulating sex-specific microglial phenotypes, at least in this model of tauopathy.

#### Sex-Related Differences in Microglial Metabolism: Evidence From Animal Models

Recent studies have shown that a shift from the homeostatic state in microglial cultures from neonatal mice (Holland et al., 2018; Rubio-Araiz et al., 2018) and in isolated microglia from aged mice (Mela et al., 2020) and APP/PS1 mice (Holland et al., 2018; Mcintosh et al., 2019) is accompanied by a shift in microglial metabolism toward a glycolytic phenotype. Factors that contribute to the metabolic shift include iron accumulation by microglia (Mcintosh et al., 2019) and IL-1β- and iron-induced activation of the glycolytic enzyme, 6-phosphofructo-2-kinase/fructose-2.6-bisphosphatase 3 (PFKFB3) (Mcintosh et al., 2019; Mela et al., 2020). These studies also showed that microglial function, specifically phagocytosis and chemotaxis, was compromised in glycolytic cells, perhaps because of the reduced efficiency of the cells in generating ATP.

Significantly, a sex-related difference shift toward glycolysis, as well as expression of genes that encode glycolytic enzymes, was observed in microglia from APP/PS1 mice and the changes were significantly greater in female APP/PS1 mice compared with males (Guillot-Sestier et al., 2021). A marked sex-related difference in microglial morphology was observed both in APP/PS1 mice and in post-mortem samples from AD patients; in both instances, microglia from males were amoeboid, with greater phagocytic capacity, compared with a preponderance of rod-shaped microglia from females where phagocytic capacity was compromised (Guillot-Sestier et al., 2021). Interestingly, an age-related decline in glucose uptake was identified in female wild type and 3xTg AD mice as assessed by [^18^F]fluorodeoxyglucose micro-positron emission tomography (FDG micro-PET) though this was not attributed to microglial metabolism but to a decrease in neuronal uptake through glucose transporter 3 (GLUT3) and a decrease in glucose utilization as indicated by decreased hexokinase II expression and activity (Ding et al., 2013).

It is well established that glucose metabolism is altered in AD, and specifically that there is a decrease in activity of several mitochondrial enzymes involved in the tricarboxylic acid cycle (Gibson et al., 2012), including α-ketoglutarate dehydrogenase but sex-related differences have not yet been assessed. TREM2 also impacts on microglial metabolism; extracellular acidification rate (ECAR) was decreased in Trem2-deficient microglia while mitochondrial mass was decreased in cells from Trem2-deficient 5XFAD mice suggesting that its loss modulates both glycolysis and mitochondrial metabolism (Ulland et al., 2017) but once again, evidence of sex-related differences have not been reported.

#### Can the Age/Disease-Related Sensitivity of Microglia Be Altered?

The sensitivity of microglia to inflammatory stimuli is age- and sex-dependent. Sala Frigerio et al. (2019) reported that microglia from female mice respond more to accumulating Aβ in the App*^NL–G–F^* mouse model of AD which shows amyloid deposition at 3 months of age while the population of microglia described as ARMs increased at an earlier age in female mice compared with males. These changes suggest a state of alertness to inflammatory stimuli in microglia from females as they age providing a possible explanation for the increased risk of diseases like AD in females. It makes sense to reduce this risk, particularly in females. Diet and exercise, which reduce microglial activation and neuroinflammation (Lynch et al., 2007; Minogue et al., 2007; Kelly, 2018; Koh et al., 2020; Mela et al., 2020) and seem to exert a greater impact on females (Barha and Liu-Ambrose, 2018; D’AMICO et al., 2020) represent a potential path toward modifying some risk. Interestingly, a high fat diet resulted in an upregulation of genes encoding the immune response and inflammation in microglia from 6-month-old female, but not male, APP/PS1 × APOE4^+/+^ mice (Nam et al., 2018) and reduced neurogenesis also in female mice, perhaps as a result of changes in microglia (Robison et al., 2020). The key message here is that, at least some lifestyle changes that can reduce disease risk particularly in the context of aging, are modulated by sex.

#### Is There Evidence That Treatments May Act in a Sex-Specific Manner?

The frank sex-related differences in microglial activation, metabolism and function that characterize amyloid pathology in models of AD suggest the possibility of sex-specific treatments. To date, analysis of the effect of modulators of change in models of AD has infrequently assessed the impact of sex although a few recent studies have highlighted the importance of this. For example, antibiotic treatment reduced Aβ plaque burden and microglial soma size, and increased microglial complexity, in 7-week-old APP*_*Swe*_*/PS1*_*L*166*P*_* (APPPS1-21) male, but not female, mice (Dodiya et al., 2019), and transcriptomic analysis indicated that antibiotics increased microglial clusters that included homeostatic genes, like *Mef2a, Junb, Bhlhe41, Fos* while decreasing clusters that included the neurodegenerative genes (e.g., *Lgals3, C1qa, C1qb, CD63*, and *Lag3*) described by Krasemann et al. (2017). Perhaps consistent with the maintenance of the homeostatic microglial signature is the finding that antibiotic treatment restored TGFβ signaling in the cortex of male mice (Dodiya et al., 2019). A separate study reported that inhibition of mGluR5 exerted beneficial effects exclusively in male APP/PS1 mice by decreasing amyloidosis and improving cognition, while no changes were observed in females (Abd-Elrahman et al., 2020). In contrast, the angiotensin receptor blocker, candesartan cilexetil, improved performance in the novel object recognition test and reduced glial activation in female 5xFAD mice crossed with mice that were homozygous for the human *APOE4* gene but had no significant effect in males (Scheinman et al., 2021).

A further study reported that dietary supplementation of 4-month old 3xTg-AD mice with the flavonoid, diosmin (0.0005%) for 6 months reduced Aβ oligomers in the brain of female mice but not males (Sawmiller et al., 2016). It also reduced tau pathology by inhibiting GSK-3α/β activity but a sex-related difference was not reported, suggesting that it specifically targets amyloid accumulation in females.

Among the many age-related changes in intracellular signaling pathways is an increase in mammalian target of rapamycin (mTOR) signaling in microglia (Keane et al., 2021), which is consistent with the finding that genes regulated by TSC2, which modulates mTOR, were decreased with age and, interestingly, to a greater extent in females compared with males (Mangold et al., 2017). The age-related increase in mTOR signaling was accompanied by an upregulation of genes like *Axl, Tlr2, Cst7* and *Spp1* (Keane et al., 2021) which we found were upregulated also in APP/PS1 mice, particularly females (Guillot-Sestier et al., 2021).

Several modulators of mTOR signaling attenuate microglial activation. Metformin and rapamycin both inhibit mTOR signaling and both attenuate microglial activation, for example following TBI (Song et al., 2015) and with age (Kodali et al., 2021). Of special interest is the sex-related differences exhibited by metformin treatment in a model of nerve injury where the effect on microglia was confined to male mice (Inyang et al., 2019). Acarbose and 17α-estradiol also inhibit the mTOR signaling pathway in males (Shen et al., 2021) but not females and both modulate microglial activation (Sadagurski et al., 2017) and both exerted a greater effect on microglial proliferation in males compared with females.

### Sex Differences in Microglia Following Acute Inflammatory Stress

#### The Effect of LPS on Microglia Is Influenced by Age and Sex

Sex-related differences in terms of inflammatory phenotype and responsiveness to inflammatory stimuli have been reported in microglia. LPS triggered a greater release of inflammatory cytokines including IL-1β from microglia isolated from the brain of P0/P1 male rats compared with females (Loram et al., 2012). Whereas LPS induced similar changes in many gene transcripts in cells from both sexes, it increased expression of genes that code for proteins involved in the immune response and, overall, triggered changes that accelerated microglial development in males that had already occurred in females (Hanamsagar et al., 2017, 2018). Responsiveness of microglia from P0 to P2 mice to IFNγ also showed a sex-related difference with cells from male mice producing more inflammatory cytokines; cell function was also altered with increased migration observed in cells from males and increased phagocytosis in cells from females (Yanguas-Casas et al., 2018).

The basis of these sex-specific changes induced by inflammatory stimuli is not known but estrogens acting through receptors ERα and ERβ, which are expressed on microglia (Johann and Beyer, 2013), are capable of modulating certain microglial responses. Studies have reported that estrogen attenuates LPS-induced production of superoxide and iNOS (Bruce-Keller et al., 2000) and expression of MHCII, CD40 and CD80, and production of inflammatory mediators (Vegeto et al., 2001; Dimayuga et al., 2005). However it is interesting that some modulators of microglial activation also exhibit sex-specific effects. For instance, 17β-estradiol attenuated the LPS-induced response in cells from P0/P1 male rats and had the opposite effect in cells from females further highlighting the sex-related differences in microglia (Loram et al., 2012). With respect to IFNγ-induced changes, palmitic acid attenuated the increase in IL-1β but this was confined to microglia from P0 to P2 male rats whereas it attenuated the IFNγ-induced increase in phagocytosis in cells from males and females (Yanguas-Casas et al., 2018).

These marked sex differences in responsiveness of microglia to inflammatory stimuli is the focus of significant attention because of the potential impact of these differences in neurodevelopmental disorders. This has been extensively reviewed (Villa et al., 2019; Bordt et al., 2020; Vanryzin et al., 2020).

Although studies to the impact of inflammatory stimuli on microglia from older mice are limited, it has been shown that responsiveness to LPS is greater in microglia from 2-month-old male, compared with female, mice (Hanamsagar et al., 2017, 2018). Similarly, LPS increased Iba1^+^ cell number and area in 3 month-old male, but not female mice (Wu et al., 2016) although administration of poly I:C to pregnant dams increased microglial activation in 2–3-month-old female offspring and exerted no effect on males (Hui et al., 2020). In contrast, LPS increased mRNA expression of inflammatory cytokines to a greater extent in tissue from aged female compared with male mice, but it is unclear if this change is microglia-specific since LPS-induced astroglial activation was also greater in females (Murtaj et al., 2019).

These data firmly establish that there are marked age- and sex-related differences in responsiveness of microglia to inflammatory stimuli and emphasize the importance of unambiguous reporting on both factors.

#### Stress Affects Microglia but Sex-Related Changes Are Influenced by Several Factors

A number of studies have set out to determine the impact of different stressors on microglia in male and female animal models, partly driven by the need to understand the greater susceptibility of females to stress-linked psychological disorders. One study compared the effect of restraint stress in 10–11-week-old male and female rats and, acute stress increased the proportion of primed microglia in male rats and had the opposite effect in females whereas chronic stress decreased the proportion of primed microglia in females and exerted no effect in males (Bollinger et al., 2016). This effect seems to be dependent on variables including age, brain area and type of stress because others reported that maternal separation decreased glial numbers in the ventral tegmental area, in P0–P14 male, compared with female, rats but no change was observed in substantia nigra (Chocyk et al., 2011). Prenatal stress also affected microglia in offspring and, specifically, sensitized the cells to inflammatory stimuli such that LPS increased Iba1 immunoreactivity in 2-month-old male mice (Diz-Chaves et al., 2013).

In a separate study, chronic unpredictable mild stress exerted no effect on microglial morphology in 3-month-old male or female rats although administration of dexamethasone to the pregnant dams increased the number and length of microglial processes in the hippocampus of females and the nucleus accumbens of males (Gaspar et al., 2021).

#### Targeting Inflammation in a Sex-Specific Manner in Stroke Is Important

Ischemic or mechanical injury to the brain triggers many changes including an acute inflammatory response that is typified by microglial activation. A recent population-based study that followed over 9 million adults over a period of about 15 years reported that females had a lower lifetime hazard of stroke than males but this ratio fluctuated with age, with premenopausal women less vulnerable and women in older age more vulnerable; overall, the outcome for females is worse (Roy-O’REILLY and Mccullough, 2014; Vyas et al., 2021). A key contributor to the increased vulnerability in older women is loss of the anti-oxidant, anti-inflammatory and neuroprotective effects of estrogens, which additionally modulate mitochondrial function (Torrens-Mas et al., 2020) and boost signaling pathways including activating phosphatidylinositol-3-kinase (PI3K) and nuclear factor erythroid 2–related factor 2 (Nrf2) (Ishii and Warabi, 2019). Like estrogens, progesterone suppresses microglial activation (Yilmaz et al., 2019) and, in middle cerebral artery occlusion (MCAO), attenuates microglial activation and neuronal damage, and improves functional outcomes (Zhu et al., 2019). However, despite the apparent beneficial effects of estrogen and progesterone in experimental models, translation into clinical use has failed to provide a convincing case for either because of lack of efficacy and/or significant side effects (Liu and Yang, 2013; Gibson and Bath, 2016).

Studies in models of stroke have highlighted sex differences in the context of neuroinflammatory changes. For instance, MCAO induced greater microglial activation as assessed by Iba1 immunoreactivity, and infarct area as revealed by diffusion-weighted imaging, in male mice compared with females. This was attributed to the fact that microglial gene expression in females is reflective of a neuroprotective/neuroreparative phenotype, compared with a more inflammatory phenotype in males (Villa et al., 2018). Interestingly infarct volume was decreased in male mice that underwent MCAO following transplantation with microglia from female mice compared with transplantation with microglia from male mice (Villa et al., 2018).

There are other factors that may partly explain the sex-related differences in response to ischemic injury, including differences in activation of the pathways that trigger poly ADP-ribose polymerase (PARP)–/caspase 3-dependent neuronal cell death as recently reviewed (Spychala et al., 2017). Significantly, neuronal cell death appears to post-date microglial activation as revealed in a study of time-related changes following transient forebrain ischemia in rats (Hamby et al., 2007). In this study, the PARP inhibitor, PJ34, completely blocked the ischemia-induced microglial activation and markedly decreased neuronal death. More recently, it was shown that post-ischemic delivery of PJ34 attenuated the MCAO-induced increases in (ipsilateral) cortical inducible nitric oxide synthase (iNOS), matrix metallopeptidase 9 (MMP9) and TNFα mRNA in male but not female mice although it decreased markers of microglial activation in both males and females (Chen et al., 2020). Consistently, in P9 mice, PJ34 reduced infarct volume in male mice but not females and this was associated with a decrease in Iba1^+^ COX2^+^ cells in male mice, which was interpreted by the authors as inflammatory microglia (Charriaut-Marlangue et al., 2018). Minocycline, which also inhibits PARP, was found to be neuroprotective in experimental stroke also in male mice, but not female mice (Li and Mccullough, 2009). This is significant because there are some promising clinical data which suggest that minocycline may be beneficial as a treatment in acute stroke (Malhotra et al., 2018), but sex-related differences, if any, remain to be explored.

Proliferator-activated receptor alpha (PPARα) agonists, which reduce inflammatory cytokine production because they decrease nuclear factor κB (NFκB) activation, also offer potential benefit and one such agonist, fenofibrate, has been shown to reduce MCAO-induced infarct volume albeit in male mice only (Dotson et al., 2016).

Additional factors that may confer relative protection after experimental stroke in female mice are the increases in IL-10 secreting CD8^+^ T cells (Banerjee et al., 2013) and an upregulation in IL-4 signaling (Rahimian et al., 2019), which are not seen in male mice. Significantly, both IL-10 and IL-4 attenuate modulate microglial activation (Lyons et al., 2007a,b; Laffer et al., 2019). The protective effect of IL-4 is consolidated by the finding that the reduced infarct volumes and neurological deficits, which are observed in females during phases of the estrus cycle when estrogen is high, are absent in IL-4-deficient female mice (Xiong et al., 2015). On the other hand, PPARγ agonists, which increase IL-4 in hippocampus (Loane et al., 2009), appear to reduce the risk of stroke (Liu and Wang, 2019).

In short, these findings demonstrate that while controlling inflammation may provide a beneficial therapeutic option in stroke, sex-related differences in treatments must be considered (a) to improve efficacy and (b) to avoid mis-interpretation of clinical findings.

#### A Focus on Sex-Related Inflammation in Traumatic Brain Injury

Men are more likely to suffer traumatic brain injury (TBI) than women, particularly work-related injury and as a result of road traffic accidents, but whether recovery is similar in males and females is unclear with mixed evidence from clinical studies, depending on factors that include age and severity of injury. A recent metanalysis of 156 studies reported worse outcomes for women than men when injury was classified as mild-moderate, and better when injury was classified as moderate-severe, although statistical power influenced this conclusion (Gupte et al., 2019).

In animal models, the evidence is also mixed and, here the confounding issues include the model used, the outcomes assessed, the age of the animals and the relative lack of focus on assessment of changes in females. Despite this, the evidence seems to tilt in favor of a protective role for estrogens in TBI as highlighted in recent reviews (Spani et al., 2018; Gupte et al., 2019); 17β-estradiol attenuates TBI-induced damage to blood vessels and blood brain barrier function, increase in intracellular calcium and mitochondrial dysfunction (Kovesdi et al., 2020).

A recent review of sex-related differences in response to injury suggests greater detrimental effects in male mice (Rahimian et al., 2019). This confirm data that demonstrated increased COX2 expression coupled with increased apoptosis (Gunther et al., 2015), and more extensive microglial activation and/or increased microglial numbers in male mice compared with female mice after either a penetrating cortical injury (Acaz-Fonseca et al., 2015) or controlled cortical impact (Doran et al., 2019).

In the latter model, a more aggressive neuroinflammatory profile was observed in cortex, thalamus and dentate gyrus of male mice during the acute and subacute phases post-injury (Villapol et al., 2017). These authors also reported an increase in Iba1^+^ cells in the brains of male mice 1–7 days after TBI, while the number of ramified microglia was increased in female mice. Sex-related changes in inflammatory cytokines were also observed with earlier and more short-lived changes in IL-1β and TNFα mRNA in the cortex of female mice contrasting with more consistent increased expression of both in cortex of males (Villapol et al., 2017). A greater infiltration of macrophages was observed in male mice following injury (Villapol et al., 2017; Doran et al., 2019) perhaps triggering the changes in microglial activation that have been described in in aged rats and APP/PS1 mice (Barrett et al., 2015a,b).

### Factors That May Contribute to Sex-Related Differences in Microglia

#### Sex Hormones

The cause(s) of the sex-related differences in microglia is far from clear with a role for sex hormones being one obvious factor and this influence has been amply demonstrated. Estrogen has profound effects on all immune cells, including microglia; receptor-induced intracellular signaling cascades as well as direct activation of estrogen response elements, which are located on promotors of immune-function genes, both exercise control over production of immune mediators (Acosta-Martinez, 2020). Thus estradiol eliminated the sex-related difference in phagocytic capacity of microglia in neonatal mice (Nelson et al., 2017) and attenuated the LPS-induced changes in microglia from adult male, but not female, rats (Loram et al., 2012). Specific age-dependent effects have been reported. For example, the microglial transcriptome, at least in terms of NFκB-regulated genes, was not markedly altered by ovariectomy or 17β-estradiol treatment in young mice (Villa et al., 2018) but in aged mice ovariectomy upregulated inflammatory molecules including TNFα and IL-1β and increased responsiveness to LPS (Benedusi et al., 2012) as recently reviewed (Villa et al., 2016; Lenz and Nelson, 2018; Acosta-Martinez, 2020).

A recent study reported that sexual dimorphic effects of 17α-estradiol and specifically showed that the age-related Iba1 immunoreactivity in hippocampus and hypothalamus was reduced by treatment of 25-month-old male but not female mice and that the effect in hippocampus was inhibited by castration, whereas ovariectomy exerted no effect (Debarba et al., 2021), which is at odds with earlier work that showed estrogens reduced the number of microglia in hippocampus of aged C57BL/6NIA female mice (Lei et al., 2003).

**FIGURE 3:**
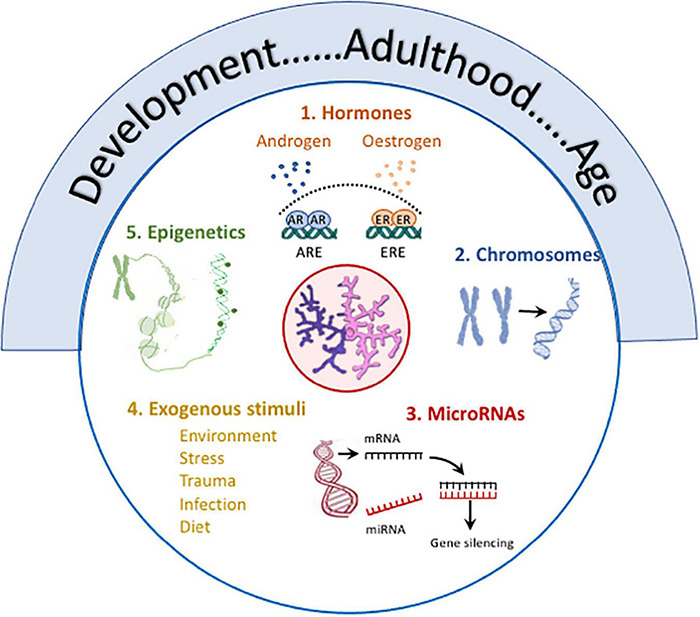
Several factors contribute to sex differences in microglia. Age, from development to old age, is marked by changes in microglia including changes in number, size, function (phagocytic, motility, antigen presentation, secretory), metabolism, expression of receptors and responses of activation stimuli, and transcriptomic signature, and age frequently impacts on other modulatory factors. **(1)** Hormones: Androgen and estrogen receptors, which are expressed in microglia, exert multiple effects with evidence of sex-specific changes. For example, estrogens trigger activation of estrogen response elements which alters immune mediator production (Acosta-Martinez, 2020), impacts on function (Nelson et al., 2017), attenuates LPS-induced changes (Loram et al., 2012) and modulates age-related inflammation (Villa et al., 2016; Lenz and Nelson, 2018; Acosta-Martinez, 2020) in a sex-specific manner. Androgen receptor expression on microglia is increased following TBI, and testosterone reduces TBI-induced reactive microgliosis and also LPS-induced cytokine production *in vitro* and microgliosis *in vivo* (see review McGovern and Baretto, 2021). **(2)** Chromosomes: X and Y chromosome genes encode for proteins with different immune functions e.g. (Klein and Flanagan, 2016; Lefevre et al., 2019). The development of the four core genotypes model have identified the contribution of chromosomes to sex-related differences in EAE (Smith-Bouvier et al., 2008) and a model of AD (Davis et al., 2020) both of which are characterized by changes in microglia. **(3)** MicroRNAs: Sex-related differences in microRNAs that regulate immune networks have been identified in microglia (Kodama et al., 2020) and depletion of Dicer in microglia leads to upregulation of genes reflecting immune activation and inflammation in males. **(4)** Exogenous stimuli: Sex-specific responses to environmental factors e.g., maternal deprivation, stress, trauma, infection, diet in microglia have been reported (see text). **(5)** Epigenetics: Epigenetic processes modulate microglia (Cheray and Joseph, 2018) and epigenetic changes [mono-methylation at lysine 4 of histone 3 (H3K4me1) and acetylation at lysine 27 of histone 3 (H3K27ac)] that support LPS-induced immune memory, controls production of inflammatory molecules in models of AD and stroke and triggers neuropathology (Wendeln et al., 2018). Specific sex-related differences in remain to be explored.

Some reports suggest that estrogen treatment improves TBI-induced changes in animal models. The presence of membrane-bound estrogen receptors on neurons may directly contribute to the increased neuronal survival observed in estrogen-treated animals following injury (Brotfain et al., 2016) (see Figure 3). However the effect of estrogens on microglia also plays a role in sparing neurons perhaps by inhibiting activation of NFκB through activation of PI3K (Crespo-Castrillo and Arevalo, 2020). Others have suggested that there is no beneficial effect of estrogen in male or female mice following TBI (Bruce-Keller et al., 2007). Progesterone receptors are also expressed on neurons and glia, and in animal models of brain injury, a protective role for progesterone in TBI has also been reported (Webster et al., 2015; Spani et al., 2018; Gupte et al., 2019) but clinical studies have failed to identify an overall beneficial effect of progesterone (Skolnick et al., 2014; Chase, 2015; Brotfain et al., 2016) and a meta-analysis of seven randomized controlled trials determined that progesterone treatment exerted no significant effect on outcomes of acute TBI in patients (Lin et al., 2015). Similarly, despite the benefits in animal models, the argument favoring the use of estrogens in clinical settings is unconvincing (Kovesdi et al., 2020). If a case is to be made for further clinical study, a focus on potential sex-specific benefits of hormone treatment would be warranted (Khaksari et al., 2018).

There is little argument that loss of estrogen at menopause is linked with increased adiposity, insulin resistance and the associated inflammation, all of which represent disease risks and confer greater risk of AD. Despite the obvious possibility that hormone replacement therapy may reduce risk, clinical trials have provided little encouragement for such a therapeutic approach. However a recent metanalysis reported that there was an overall beneficial effect in the 16 studies that were assessed (Song et al., 2020) although there are confounding issues that remain to be addressed one of which is to establish whether there is a critical window for hormone therapy in AD, as has been suggested (Song et al., 2020; Wu et al., 2020).

A focus on analysis of change in microglia during the estrus cycle would deepen our understanding of the impact of hormonal control but there is a paucity of information linking the estrus cycle with microglial status. However it has been reported that stress-induced IL-1β expression in the paraventricular nucleus, which may reflect microglia status, is attenuated during metestrus (Arakawa et al., 2014). Additionally, a transcriptomic analysis of changes in the medial prefrontal cortex across the estrus cycle identified a vast array of differences during the cycle that were markedly more profound than differences between samples from males and females. The differences reflect changes in genes that code for proteins which support multiple cell functions from metabolism to cell signaling. Although the authors discussed the changes in the context of neuronal function, many including cell signaling, cell adhesion and membrane function may potentially also reflect changes in microglia (Duclot and Kabbaj, 2015).

#### Other Factors

Clearly chromosomal makeup contributes significantly to sex-related differences given that genes on X and Y chromosomes encode for proteins with different immune functions. This is well illustrated by the finding that LPS-induced inflammatory cytokine production from monocytes obtained from male subjects is greater than from females and also from individuals with Klinefelter syndrome, who are phenotypically male but carry an extra X chromosome (Klein and Flanagan, 2016; Lefevre et al., 2019).

The development of recent models have triggered an increase in understanding the contribution of chromosomes to sex-related differences in some diseases. For example. EAE induced by proteolipid protein was more severe in SJL castrated male mice that were XX *Sry* compared with XY^–^*Sry* and also in ovariectomized female XX compared with XY^–^ mice (Smith-Bouvier et al., 2008). The authors reported that the relative protection was associated with higher production of Th2 cytokines in XY^–^ mice. Furthermore adoptive transfer of lymph node cells from ovariectomized female XX into WT females induced more severe disease than cells from and XY^–^ mice and this was associated with evidence of enhanced inflammation as suggested by an increase in CD45^+^ cells in the thoracic spinal cord.

A similar approach was used to evaluate the contribution of chromosomes to mortality in a model of AD, given that the longevity of women with AD is greater than men. It was reported that the addition of an X chromosome conferred resilience. Specifically, XY-APP mice died earlier than XX-APP mice of either gonadal phenotype and cognitive testing revealed that performance of XY-APP mice was poorer (Davis et al., 2020). Both studies clearly demonstrate the impact of chromosomal make-up in these 2 disease models, the pathology of which includes inflammation, but neither directly assess details of inflammatory change or changes in microglia. There is a paucity of literature exploring the impact of sex chromosomes on microglia.

There is little doubt that microglia are subject to epigenetic control (Cheray and Joseph, 2018) and indeed epigenetic changes underpin LPS-induced immune memory in microglia in the APP23 model of AD and trigger neuropathology (Wendeln et al., 2018) although sex-related differences were not explored. Multiple sex-related differences indicative of epigenetic changes have been described in the brain; for example, DNA methylation and methylated CpG sites are more pronounced in female brains compared with males, expression of ERa is modulated by promoter modulation and the effect of maternal behavior induces differential epigenetic changes in males and females that may impact on behaviors/pathologies in later life (Ratnu et al., 2017). However, epigenetic changes that specifically target microglia in a manner that may explain sex differences remain to be identified.

Among other factors that influence microglial phenotype and function are microRNAs (miRNA) which have multiples roles including regulation of immune networks, and miRNA-seq analysis in microglia has revealed sex-related differences (Kodama et al., 2020). Depleting Dicer in microglia, which markedly reduced miRNA, differentially affected the transcriptome in microglia from male and female mice and led to upregulation of genes reflecting immune activation and inflammation in males.

Another factor that may impact on microglial activation is infiltration of immune cells. In this context, it has been shown that infiltration of macrophages into the brain of aged rats and APP/PS1 mice is associated with increased inflammation (Barrett et al., 2015a,b), while macrophages from both models are sensitized to inflammatory stimuli suggesting that they contribute to the inflammation. Importantly, flow cytometric analysis has revealed that the number of CD11b^+^/CD45*^high^* macrophages was greater in the brain of 12-month-old female, compared with male APP/PS1, mice (Unger et al., 2018) suggesting that the more inflammatory microglia observed in older female mice might, at least in part, result from macrophage infiltration. In contract, in adult mice, infiltration of macrophages following traumatic brain injury was greater in males than females (Doran et al., 2019) paralleling the greater microglial activation and evidence of neuronal damage, at least in the early stages following injury.

## Conclusion

The focus of this review has been on conditions that are unified by the sex differences in microglial activation and neuroinflammation that characterize them and contributes to their pathogenesis. The question therefore arises as to whether sex-specific therapies, perhaps particularly those targeting neuroinflammation and microglial activation, will provide a step toward precision medicine.

A challenge is to address the under-representation of women in clinical trials which, although improving, persists (Labots et al., 2018; Steinberg et al., 2021). A report of 38 studies conducted on drugs approved by the FDA between 2000 and 2009 highlighted that a gender-imbalance occurred in phase I but not phase II and III trials (Labots et al., 2018) but an analysis of 2020 clinical trials between 2000 and 2020 revealed that sex bias continues to exist in clinical trials (Steinberg et al., 2021) and a recent study highlighted the important fact that women are less likely to meet pre-screening criteria for inclusion in clinical trials in AD in Spain and education was a key factor (Rosende-Roca et al., 2021).

Sex-related differences in absorption and excretion, distribution and metabolism of drugs have been described and more adverse drug reactions have been reported in females compared with males (Whitley and Lindsey, 2009) highlighting sexual dimorphic responses to drugs, notably drugs that affect the CNS. In the context of AD, a recent review and metanalysis reported that only 7 out of the 56 randomized clinical trials in AD included reported sex-stratified results and concluded (Martinkova et al., 2021) and the relative lack of data on sex-related differences in drug efficacy has been noted (Ferretti et al., 2018). In the light of this, a call has been made for specific analysis of gender effects on drug treatments in AD (Schwartz and Weintraub, 2021) but this cannot be confined to AD and the principle should apply broadly, but particularly in conditions where clear sex-related differences in disease incidence exists.

## Author Contributions

The author confirms being the sole contributor of this work and has approved it for publication.

## Conflict of Interest

The author declares that the research was conducted in the absence of any commercial or financial relationships that could be construed as a potential conflict of interest.

## Publisher’s Note

All claims expressed in this article are solely those of the authors and do not necessarily represent those of their affiliated organizations, or those of the publisher, the editors and the reviewers. Any product that may be evaluated in this article, or claim that may be made by its manufacturer, is not guaranteed or endorsed by the publisher.
